# The impact of disease time, cervical alignment and range of motion on cervical vertebral Hounsfield unit value in surgery patients with cervical spondylosis

**DOI:** 10.1186/s13018-023-03675-y

**Published:** 2023-03-10

**Authors:** Zhiqiang Wang, Zaowei Zhong, Haoyu Feng, Jun Mei, Xiaoning Feng, Beiyang Wang, Lin Sun

**Affiliations:** 1 Department of Orthopedics, Shanxi Bethune Hospital, Shanxi Academy of Medical Sciences, Tongji Shanxi Hospital, Third Hospital of Shanxi Medical University, Taiyuan, 030032 China; 2 Tongji Hospital, Tongji Medical College, Huazhong University of Science and Technology, Wuhan, 430030 China

**Keywords:** Cervical vertebral, Bone mineral density, Hounsfield unit, Disease time, Flexion C2-7 Cobb angle

## Abstract

**Study design:**

This study was a retrospective review.

**Objective:**

Bone mineral density (BMD) at the surgical site is associated with complications of surgical internal fixation, and it is very important to study the cervical BMD of patients with cervical spondylosis who need surgery and the related factors that affect cervical BMD. It is still unclear about the age-related influence of disease time, cervical alignment and range of motion (ROM) on cervical vertebral Hounsfield unit (HU) value.

**Methods:**

This retrospective study was conducted on patients who underwent cervical surgery at one institution between January 2014 and December 2021. Age, sex, body mass index (BMI), disease type, comorbidities, neck pain, disease time, C2-7 Cobb angle (CA), cervical ROM and the C2-C7 vertebral HU value were recorded. The association between cervical HU value and each parameter of interest was assessed using the Pearson correlation coefficient. Multivariable linear regression analysis was performed to examine the relative influence of the multiple factors on cervical vertebral HU value.

**Results:**

Among patients younger than 50 years old, the HU value of the cervical vertebral in females was higher than that of males, but after the age of 50 years, the value of females was lower than that of males and decreased significantly after 60 years old. In addition, cervical HU value was significantly correlated with the disease time, flexion CA and ROM. Our age-related subgroup of multivariate linear regression analyses shows that disease time and flexion CA negatively affected the C6-7 HU value in more than 60-year-old males and in more than 50-year-old females.

**Conclusions:**

Disease time and flexion CA were negatively affecting the C6-7 HU values in more than 60-year-old males and in more than 50-year-old females. More attention should be paid to bone quality in cervical spondylosis patients with longer disease time and larger convex of flexion CA.

## Introduction

As the elderly population continues to increase and lifestyle changes, the incidence of cervical spondylosis has increased and patients requiring surgical treatment also raised [1]. Bone mineral density (BMD) at the surgical site is associated with complications of surgical internal fixation, such as screw pullout, titanium mesh cage (TMC) subsidence and pseudarthrosis [2, 3]. Therefore, it is very important to study the cervical BMD of patients with cervical spondylosis who need surgery and the related factors that affect cervical BMD.

Dual-energy X-ray absorptiometry (DXA) is currently the gold standard for measuring BMD and diagnosing osteoporosis [4]. However, the BMD is measured in the lumbar spine and femoral neck, osteoporotic fracture occurs in 10% of patients with a normal T value, and the T value of degenerative diseases cannot accurately reflect the BMD of the patient at the surgical site [5, 6]. At present, the Hounsfield unit (HU) value by computerized tomography (CT) examination for BMD measurement has been applied to the spine. Several reports have shown that the HU value of the cervical vertebral was closely related to BMD and varied significantly among vertebral levels [7–9]. Our previous research suggested that patients with a low HU value of the inferior vertebral body of the operative segment have a higher risk of TMC subsidence in the early postoperative period after anterior cervical corpectomy and fusion [10]. In addition, cervical CT is a routine preoperative examination for cervical spine surgery, so the HU value of cervical spine can be easily obtained without additional imaging examination, which reduces the radiation exposure of patients. Therefore, measurement of vertebral HU values at the surgical level may be a more accurate method for assessing regional BMD and predicting complications.

BMD is affected by many factors such as age, sex, weight, smoking, genetics and exercise [11–13]. However, there are few studies on the impact of cervical BMD. Cervical spondylosis with degenerative changes can predispose to cervical spine pathologies such as cervical kyphosis, cervical stiffness and neck pain. These may affect the alignment of the cervical spine and subject the cervical vertebral to a variety of loads. It has been reported that even during static neutral positions and simple planar movements such as flexion/extension, head weight and neck muscle engagement can create variable loads between the vertebral levels [14, 15]. The length of the disease time also affects the pathologies of cervical vertebrae. However, it is still unclear about the age-related influence of disease time, cervical alignment and ROM on cervical vertebral HU value in surgery patients with cervical spondylosis.

In this study, we measured the HU value based on preoperative cervical CT and analyzed general patient information and radiologic evaluation, and we clarified the age-related influence of disease time, cervical alignment and ROM on cervical vertebral HU value.

## Materials and methods

### Patient population

This was a retrospective study and was approved by the Ethical Committee of our hospital. We reviewed cervical spondylosis patients undergoing surgery in our hospital between January 2014 and December 2021 in our orthopedic department. The basic information of patients was inquired according to the case system. The disease time was determined according to the patient's complaint in the case system. In this work, the inclusion criteria were as follows: [1] diagnosis of cervical spondylosis; [2] patients with preoperative cervical CT and X-ray within 1 week before surgery; and [3] accept cervical surgery at our orthopedic department. The exclusion criteria were as follows: [1] patients with spine infection, spine tumor, spine trauma and metabolic bone disease; [2] merged cervical spine posterior longitudinal ligament ossification or multiple osteosclerosis; [3] long-term use of hormones or combined with immune diseases; [4] patient with nervous system disorders such as demyelinating disease; [5] a history of previous spinal surgery; [6] diagnosed with osteoporosis and treated with medication; and [7] incomplete radiologic data.

### HU measurements

**Fig. 1:**
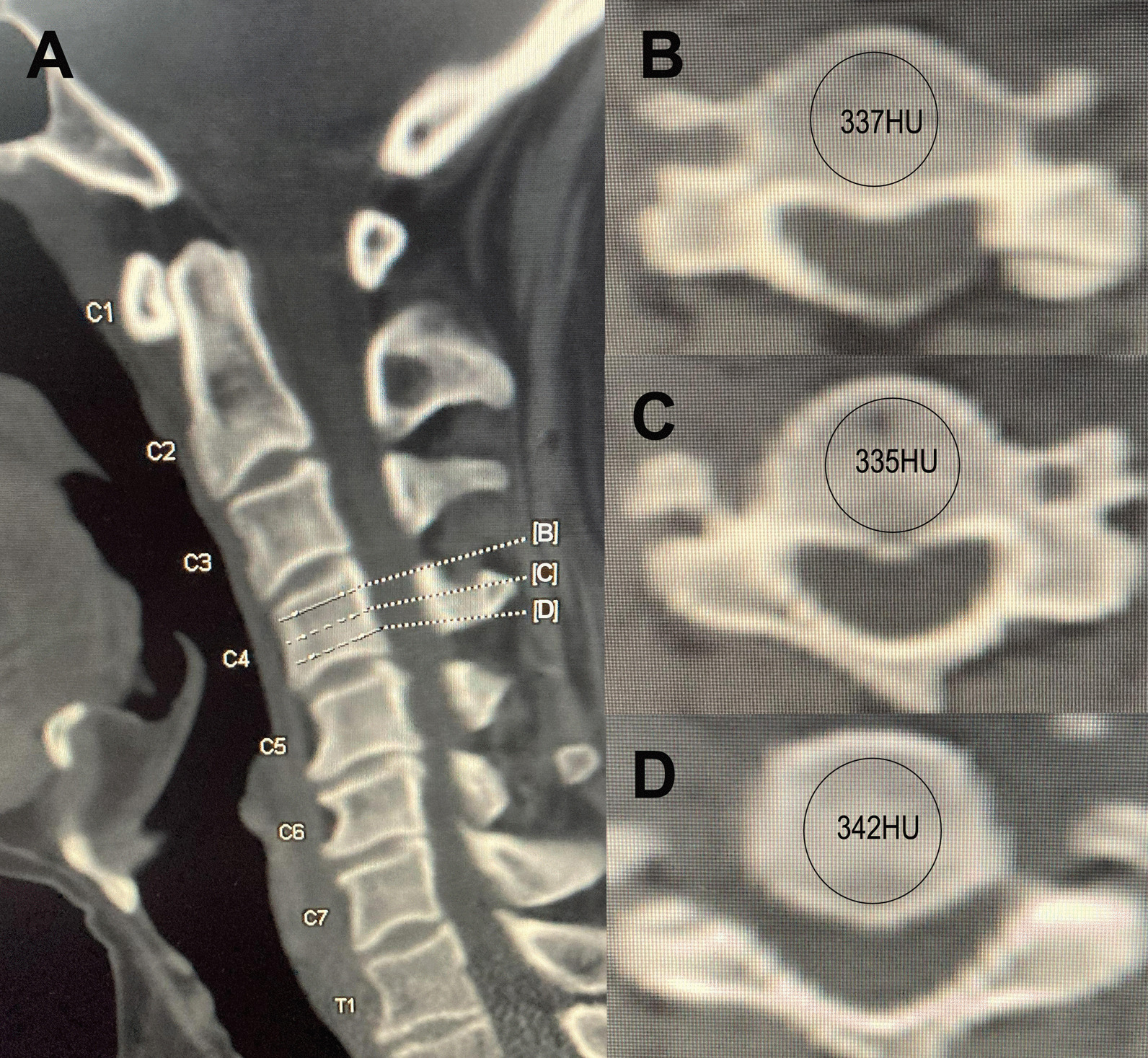
Midsagittal (**A**) and axial CT images demonstrating the measurement of vertebral HU value on the axial plane inferior only to the superior endplate (**B**), middle of the vertebral (**C**) and axial plane superior only to the inferior endplate (**D**) (the first letter C stands for cervical vertebra, which consists of seven segments from top to bottom, denoted by C1-C7, the same as T1)

All patients in our hospital underwent three-dimensional reconstructive cervical CT (PHILIPS, Brilliance, slice thickness 1.5 mm, distance 1.5 mm, tube voltage 120 kV) within 1 week before surgery. We use the picture archiving and communication system (PACS) measurement of the C2-C7 HU value. HU values were measured using CT scans according to a previously described method [16]. The average HU values of each vertebral body were based on the axial plane inferior only to the superior endplate, middle of the vertebral body and axial plane superior only to the inferior endplate. The HU value was measured by placing the largest elliptical region of interest (ROI) at the mid-vertebral body, and the ROI was chosen to include as much trabecular bone as possible and to avoid cortical bone and heterogeneous areas, such as cortical bone margins, osteophytes and osteosclerosis. The average of HU values measured from the three ROIs was regarded as the HU for the individual vertebral (Fig. 1).

### Radiographic measurements

**Fig. 2:**
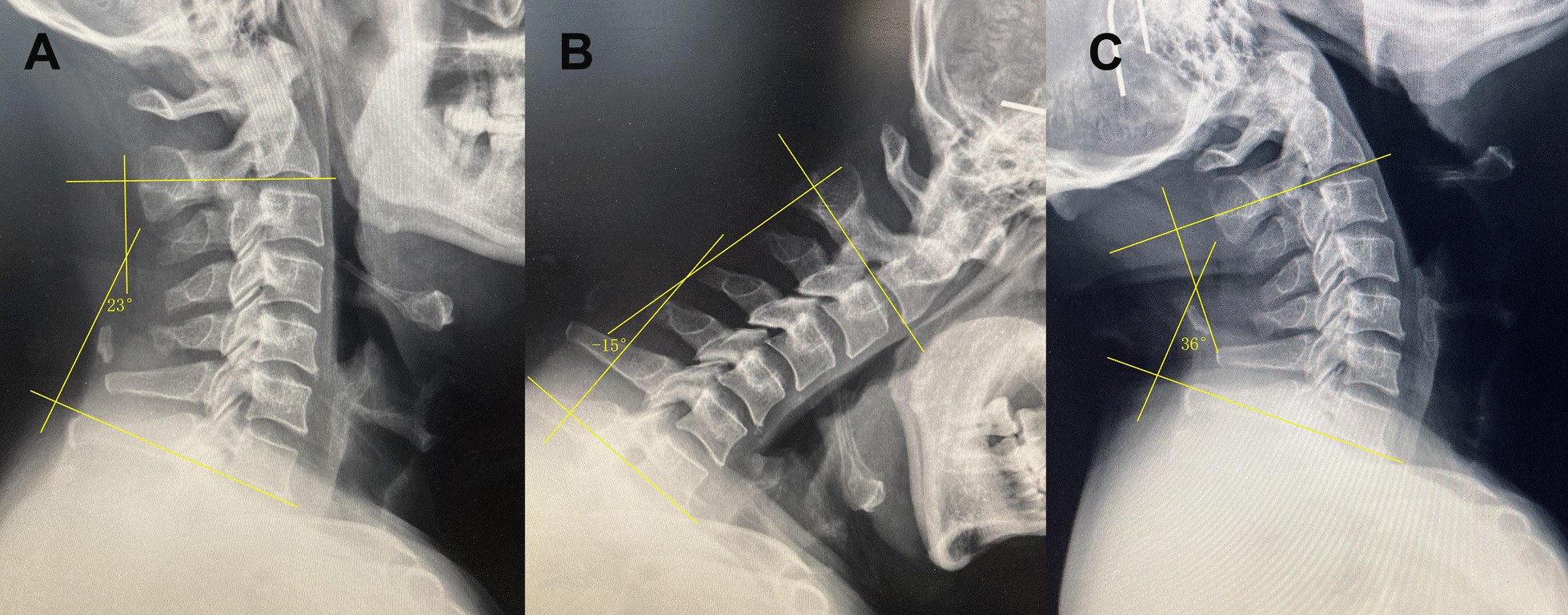
Radiologic parameters on plain radiographs of neutral (**A**), flexion (**B**) and extension (**C**) included C2-7 Cobb angle (CA)

Radiologic parameters on plain radiographs of neutral, flexion and extension included C2-7 Cobb angle (CA). Lateral X-ray images were obtained while the patients were standing and in a straightforward position. Flexion and extension radiography was performed with the neck in maximum flexion and extension position. The CA was defined by the Cobb angle formed between the lower endplate of C2 and C7. On the cervical spine dynamic X-ray film, parallel lines were drawn along the lower endplate of C2 and the lower endplate of C7, respectively, and the angle between the two parallel lines was the cervical Cobb angle. (Convex is a positive value, kyphosis is a negative value.) Cervical ROM = extension CA –flexion CA (Fig. 2).

### Statistical analysis

Statistical analysis was conducted using SPSS software (version 26.0, USA). After an agreement was reached between the observers, each parameter was independently measured twice by 2 orthopedic spine surgeons. Categorical variables are presented as absolute numbers and percentages, and continuous variables are presented as mean ± standard deviations. All data are normally distributed. An analysis of variance (ANOVA) was performed to identify different cervical HU values among inter-group comparisons. The independent sample t-test was used for intra-group comparison. The association between cervical HU value and each parameter of interest was assessed using the Pearson correlation coefficient (*r*). The strength of the correlation was classified as negligible (0.00–0.10), weak (0.10–0.39), moderate (0.40–0.69), strong (0.70–0.89) and very strong (0.90–1.00)[17]. Multivariate linear regression analysis was carried out to examine the age- and sex-related influence of disease time, cervical alignment and ROM on the cervical HU value. *P* < 0.05 was considered to indicate a statistically significant difference.

## Results

### Demographic characteristics

**Fig. 3:**
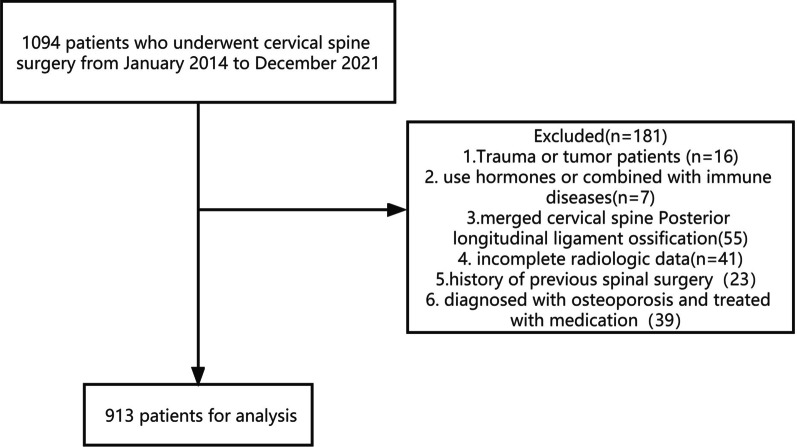
A flowchart of patients was included in the study

We reviewed 1094 cervical spondylosis patients who underwent surgery between January 2014 and December 2021, and a total of 913 patients met the inclusion criteria (Fig. 3), including 563 males and 350 females, with a mean age of 55.98 ± 10.61 years (range, 26–85 years). The average disease times were 22.64 ± 30.98 months in male and 31.89 ± 38.59 months in female. Table 1 illustrates the demographic characteristics of the subjects in this study.

**Table 1:** Demographic characteristics

Variable	Mean/N	SD/%
Age (years)	55.98	10.61
< 40	66	7.22
40–50	215	23.55
50–60	304	33.30
60–70	254	27.82
> 70	74	8.11
Sex		
Male	563	61.66
< 40	48	5.26
40–50	125	13.69
50–60	180	19.71
60–70	163	17.85
> 70	47	5.15
Female	350	38.34
< 40	18	1.97
40–50	90	9.86
50–60	124	13.58
60–70	91	9.97
> 70	27	2.96
Body mass index (kg/m2)	24.91	5.63
Combined neck pain	315	34.50
Disease time (months)		
Male	22.64	30.98
Female	31.89	38.59
Comorbidities	330	36.14
Hypertension	169	18.51
Diabetes	59	6.46
Cardiac disease	25	2.74
Merged	77	8.43
Disease type		
Radiculopathy	90	9.86
Spondylosis	657	71.96
Mixed	166	18.18
HU value		
C2	371.86	83.23
C3	360.13	76.28
C4	372.50	79.36
C5	360.93	79.79
C6	319.35	73.40
C7	274.08	61.27

Values are the mean and standard deviation or number and percentage

### The trend of C2-C7 vertebral HU value

**Fig. 4:**
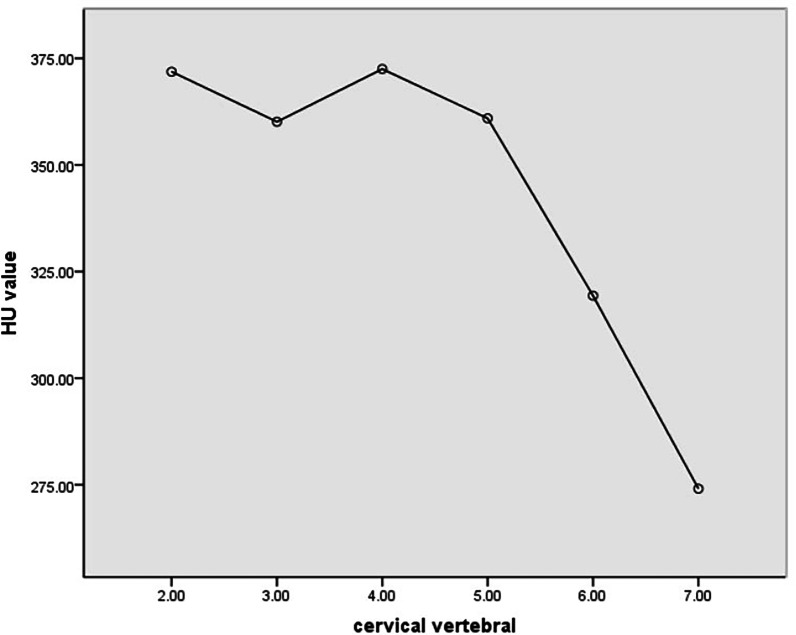
Trend of C2-C7 vertebral HU value. C2-C5 HU values are maintained at a baseline, the decrease was obvious at C6 and the C7 vertebral had the minimum HU value

The cervical HU value was different at each level (Fig. 4 and Table1). C2-C5 HU values are maintained at a baseline (ranging from 360.13 ± 76.28 to 372.5 ± 79.36). The decrease was obvious at C6 (319.35 ± 73.40), and the C7 vertebral had the minimum HU value (274.08 ± 61.27). Therefore, according to the trend of HU value of C2-C7 cervical vertebral, we divided cervical vertebral HU value into upper cervical vertebral (average HU value of C2-5 vertebral), lower cervical vertebral (average HU value of C6-7 vertebral) and overall cervical vertebral (C2- C7 vertebral average HU value).

### Influence of age and sex on cervical vertebral HU value

We divided the patients into 5 groups by 10 years old; since we collected fewer cervical surgery patients younger than 30 years and older than 80 years, we included these patients into the less than 40 years groups and more than 70 years groups.

**Fig. 5:**
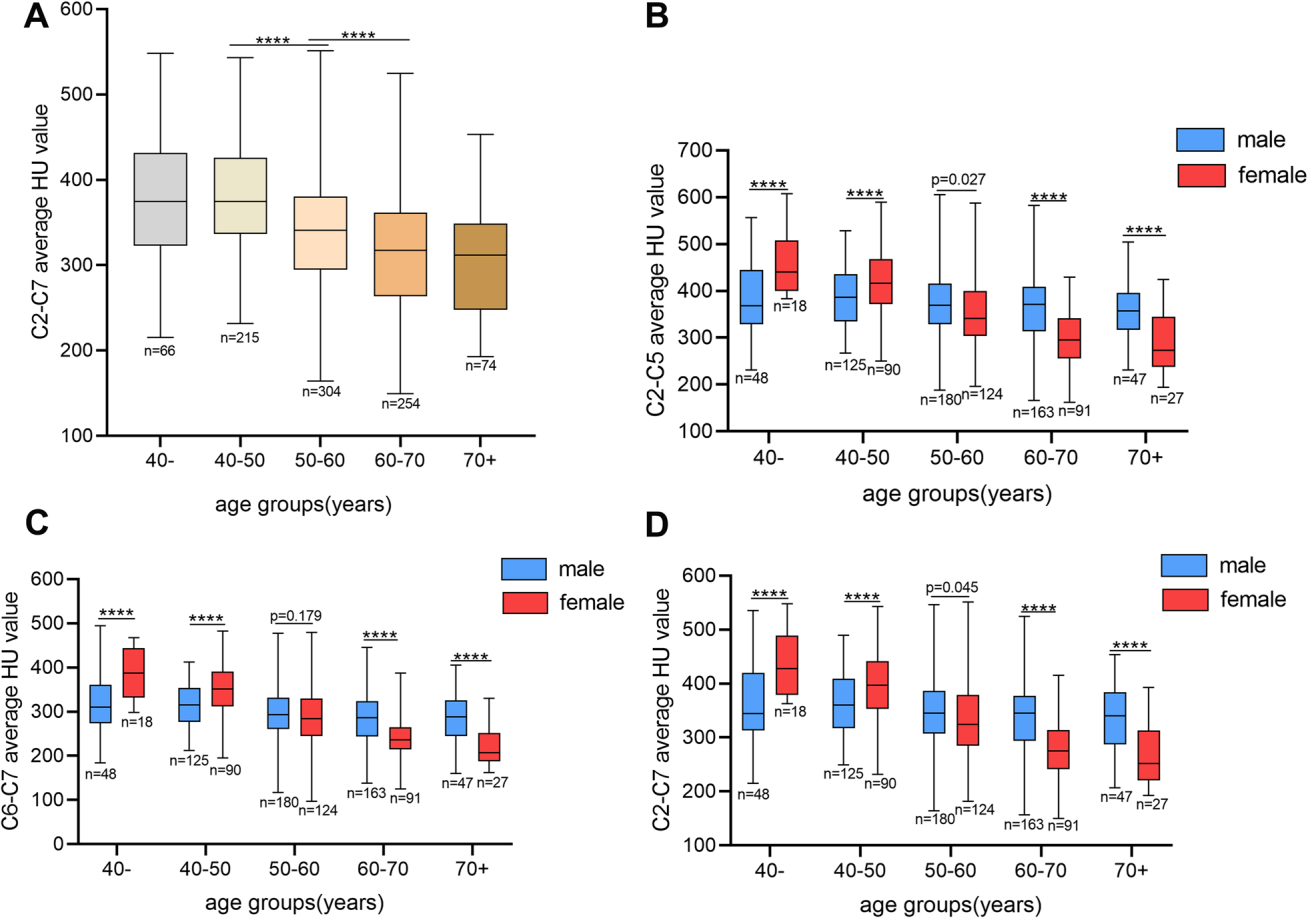
Box plots showing the trend of cervical spine HU value with age groups (**A**) and the median value and range of C2-5 (**B**), C6-7 (**C**) and C2-7 (**D**) average HU value by age and sex (****indicates significant difference between males and females, *p* < 0.0001)

First, we observed the cervical vertebral HU value changes with age, and the results suggest that the less than 40 age group was no statistical difference compared to the 40–50 age group (*P* = 1.000, 384.11 ± 73.88 vs. 377.24 ± 60.09), between 60 and 70 age group and the more than 70 age group were also no statistical difference (*P* = 1.000, 317.22 ± 67.35 vs. 305.86 ± 65.97), and the 50–60 age group (340.86 ± 68.63) was significantly different compared to less than 50 and more than 60 age groups (*P* < 0.0001) (Table 2 and Fig. 5A).

**Table 2:** Cervical HU values by age and sex grouping

Ages	Sex	Number of cases	C2	C3	C4	C5	C6	C7	Average C2-C5	Average C6-C7	Average C2-7
< 40		66	404.52 ± 81.25	400.02 ± 79.69	414.05 ± 80.54	403.96 ± 79.27	368.75 ± 79.04	313.35 ± 64.93	405.64 ± 78.07	341.05 ± 70.28	384.11 ± 73.88
	M	48	385.99 ± 80.57	383.69 ± 77.20	394.95 ± 77.99	381.44 ± 72.59	350.88 ± 77.29	295.92 ± 61.79	386.52 ± 75.13	323.40 ± 67.43	365.48 ± 71.04
	F	18	453.94 ± 61.15	443.56 ± 71.02	464.98 ± 64.87	464.02 ± 64.85	416.43 ± 63.75	359.83 ± 49.21	456.63 ± 62.58	388.13 ± 55.60	433.79 ± 57.65
40–50		215	404.18 ± 70.41	390.36 ± 66.32	369.96 ± 77.40	398.70 ± 68.53	356.14 ± 62.29	307.45 ± 50.53	399.96 ± 64.81	331.80 ± 54.72	377.24 ± 60.09
	M	125	392.07 ± 65.81	378.98 ± 64.03	393.02 ± 68.37	382.79 ± 65.19	339.89 ± 57.55	292.82 ± 46.10	386.72 ± 61.96	316.36 ± 49.96	363.26 ± 56.51
	F	90	420.99 ± 73.45	406.17 ± 66.55	425.49 ± 65.20	420.80 ± 67.24	378.70 ± 61.88	327.78 ± 49.60	418.36 ± 64.50	353.24 ± 54.07	396.65 ± 59.81
51–60		304	373.06 ± 81.65	358.55 ± 74.25	347.29 ± 76.55	358.02 ± 78.60	315.43 ± 68.61	270.18 ± 57.66	364.90 ± 74.47	292.80 ± 61.86	340.86 ± 68.63
	M	180	382.44 ± 78.58	364.95 ± 71.96	377.53 ± 74.49	366.05 ± 78.33	320.99 ± 68.48	272.54 ± 56.06	372.74 ± 71.98	296.76 ± 60.80	347.42 ± 66.38
	F	124	359.44 ± 84.38	349.25 ± 76.80	358.97 ± 80.48	346.36 ± 77.83	307.36 ± 68.28	266.75 ± 59.99	353.51 ± 76.81	287.06 ± 63.17	331.36 ± 70.95
61–70		254	344.03 ± 83.94	336.13 ± 73.98	347.29 ± 76.55	333.62 ± 73.15	291.53 ± 67.04	250.73 ± 55.01	340.27 ± 73.56	271.13 ± 59.40	317.22 ± 67.35
	M	163	367.85 ± 81.94	358.33 ± 71.21	371.88 ± 74.00	357.68 ± 71.46	311.15 ± 66.88	263.88 ± 56.03	363.93 ± 70.51	287.52 ± 59.64	338.46 ± 65.16
	F	91	301.37 ± 69.68	296.35 ± 61.35	303.26 ± 59.67	290.52 ± 54.05	256.38 ± 51.35	227.18 ± 44.51	297.87 ± 58.58	241.78 ± 46.46	279.18 ± 53.19
> 70		74	339.47 ± 79.02	325.62 ± 70.32	333.25 ± 71.21	318.55 ± 75.27	280.02 ± 68.92	238.24 ± 58.60	329.22 ± 70.68	259.13 ± 61.79	305.86 ± 65.97
	M	47	361.12 ± 70.19	347.75 ± 63.80	356.41 ± 65.77	345.28 ± 70.59	310.25 ± 61.87	255.84 ± 59.24	352.64 ± 63.51	283.05 ± 58.40	329.44 ± 59.69
	F	27	301.79 ± 80.58	287.09 ± 65.23	292.93 ± 62.65	272.02 ± 59.59	227.40 ± 45.12	207.59 ± 43.55	288.46 ± 64.61	217.49 ± 43.04	264.80 ± 56.20
Sum		913	371.86 ± 83.23	360.13 ± 76.28	372.50 ± 79.36	360.93 ± 79.79	319.35 ± 73.40	274.08 ± 61.27	366.36 ± 76.44	296.71 ± 65.91	343.14 ± 71.45

Values are the mean ± standard deviation. M: male; F: female

Next, we study the effect of sex in different age groups on C2-5, C6-7 and C2-7 average HU values. In less than 50 age groups, males had lower total HU values than females (*P* < 0.0001, in the less than 40 age group, 365.48 ± 71.04 vs. 433.79 ± 57.65, in the 40–50 age group, 363.26 ± 56.51 vs. 396.65 ± 59.81), whereas in the 50–60 age group, HU values in males started to be higher than in females, the difference was slightly statistically significant in C2-5 (*P* = 0.027, 372.74 ± 71.98 vs. 353.51 ± 76.81) and C2-7 (*P* = 0.045, 347.42 ± 66.38 vs. 331.36 ± 70.95), and there was no statistical difference in C6-7 (*P* = 0.179, 296.76 ± 60.80 vs. 287.06 ± 63.17). It indicates that the HU value of the C6-7 may have more obvious changes. In more than 60 age groups, males had higher cervical HU values than females (*P* < 0.0001, in the 60–70 age group, 338.46 ± 65.16 vs. 279.18 ± 53.19, in more than 70 age group, 329.44 ± 59.69 vs. 264.80 ± 56.20) (Table 2 and Fig. 5B, D).

**Fig. 6:**
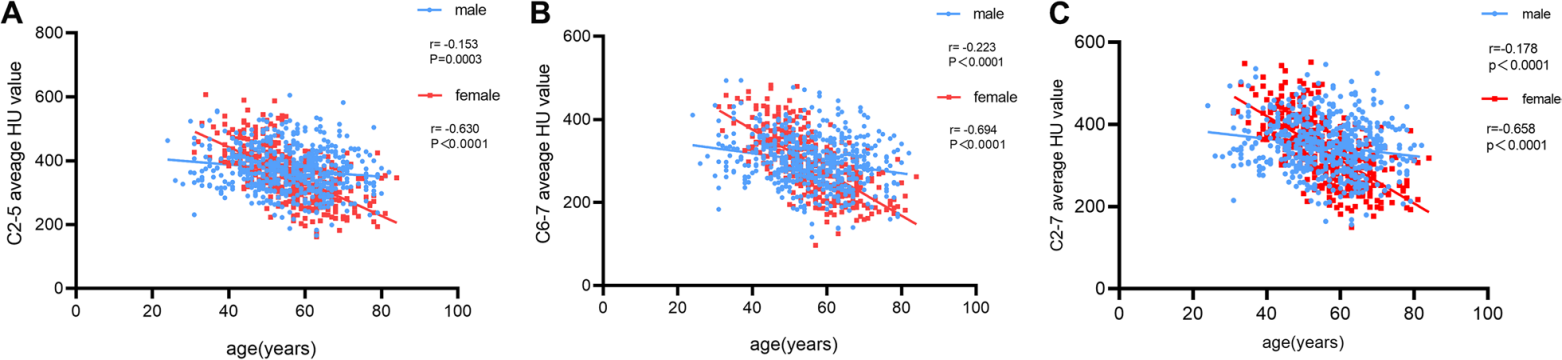
Correlation between age and C2-5 (**A**), C6-7 (**B**) and C2-7 (**C**) average HU value in males and females

Then, according to correlation analysis, there were weak but significant negative correlations between age and C2–5 (*r* = − 0.153, *p* < 0.0001), C6-7 (*r* = − 0.223, *p* < 0.0001) and C2-7 (*r* = − 0.178, *p* < 0.0001) average HU value in males. However, in females, there was moderate statistical correlation between age and C2-5 (*r* = − 0.630, *p* < 0.0001) and C2-7 (*r* = − 0.658, *p* < 0.0001) average HU value. Importantly, there were strong correlations between age and C6-7 (*r* = − 0.693, *p* < 0.0001) average HU value in females (Fig. 6).

### Influence of disease time on cervical vertebral HU value

**Fig. 7:**
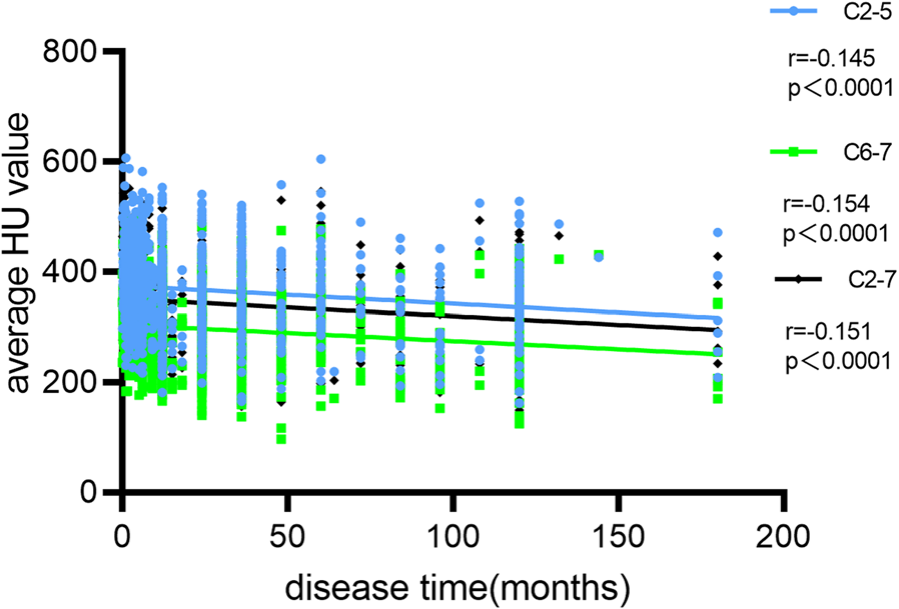
Correlation analysis between disease time and C2-5, C6-7 and C2-7 vertebral HU value

We found that C2-5, C6-7 and C2-7 average HU values decreased with the increased disease time and there were weak but significant negative correlations between disease time and C2–5 (*r* = − 0.145, *p* < 0.0001), C6-7 (*r* = − 0.154, *p* < 0.0001) and C2-7 (*r* = − 0.151, *p* < 0.0001) average HU values (Fig. 7).

### Influence of cervical alignment and ROM on cervical vertebral HU value

**Fig. 8:**
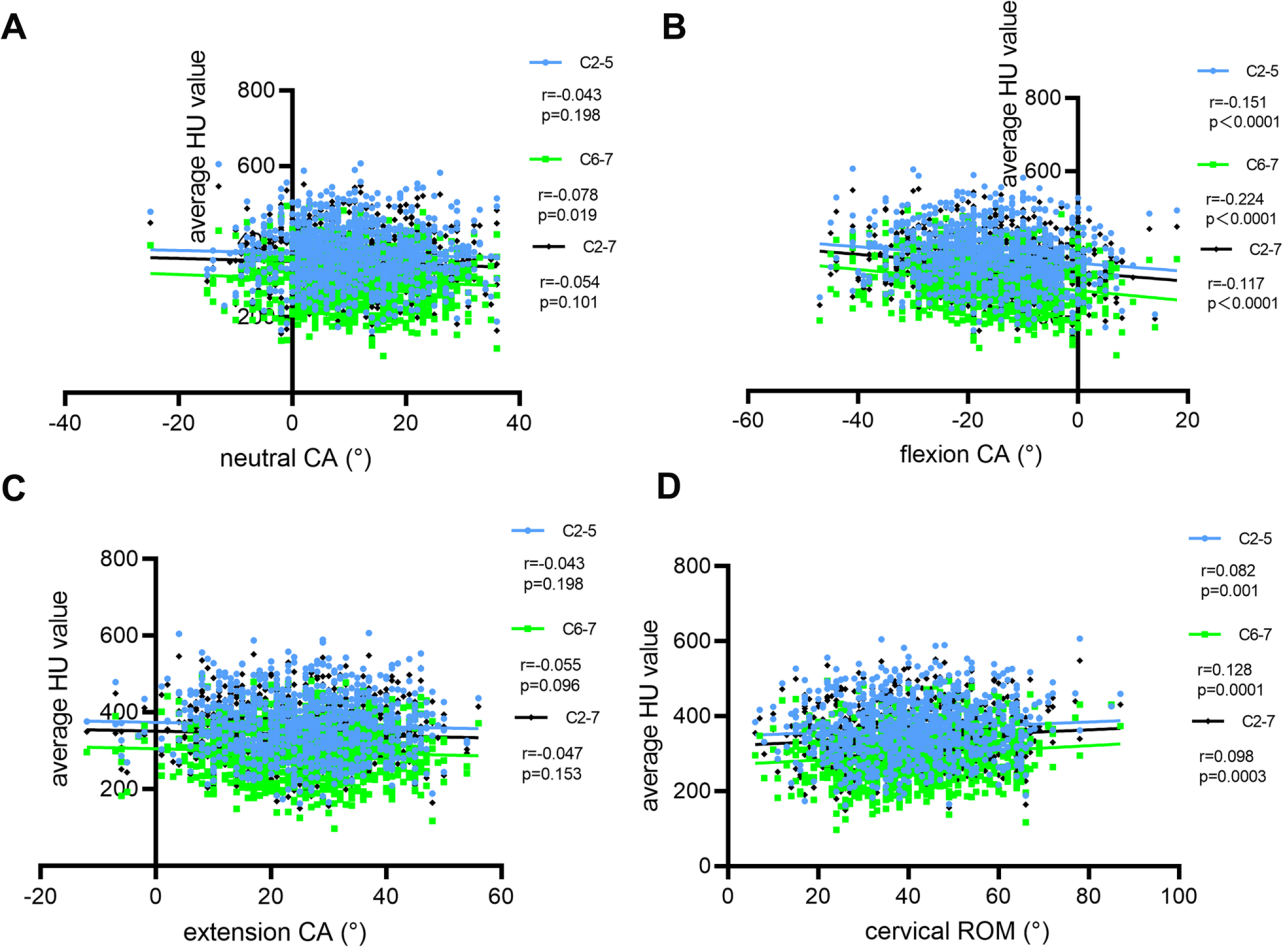
Correlation analysis between neutral (**A**), flexion (**B**), extension (**C**) CA, cervical ROM (**D**) and cervical HU value

There was negligible but significant correlation between neutral CA and C6-7 (*r* = − 0.078, *p* = 0.0019) average HU value. Between extension CA and cervical HU value were no significant correlations (*p* > 0.05, respectively). There were significant positive correlations between cervical ROM and cervical HU value, but only the C6-7 (*r* = 0.128, *p* = 0.0001) average HU value has weak correlations. However, there were weak but significant correlations between flexion CA and C2–5 (*r* = -0.151, *p* < 0.0001), C6-7 (*r* = − 0.224, *p* < 0.0001) and C2-7 (*r* = − 0.117, *p* < 0.0001) average HU value (Fig. 8).

### Results of subgroup analysis

We next performed subgroup analyses according to age and sex, and there were no statistically significant differences in the cervical HU values between the groups less than 40 years old and 40–50 years old and between the groups aged more than 70 years and 60–70 years. Therefore, we divided patients into three subgroups: the less than 50 years old, the 50–60 years old and the more than 60 years old. We performed subgroup analyses to study the influence of disease time, flexion CA and cervical ROM on the HU value of the cervical.

As presented in Table 3, C2-5, C6-7 and C2-7 average HU value is the dependent variables, and then, disease time, flexion CA and cervical ROM were taken together as independent variables. Adjusted for age (not for age subgroup analyses), BMI, neck pain, comorbidities and disease type, our age-related subgroup of multivariate linear regression analyses shows that disease time and flexion CA negatively affected C6-7 HU value in more than 60-year-old males and in more than 50-year-old females. In other words, the C6-7 average HU value decreases with the longer disease time and larger convex of flexion CA, whereas disease time, flexion CA and cervical ROM were not affected cervical HU value in less than 60 years old in males and less than 50 years old in females.

**Table 3:** Results of multivariate linear regression analyses

Age group	Dependent variable	Independent variables	Male				Female			
			B	SE	*β*	*p* value	B	SE	*β*	*p* value
All	C2-5 HU value	Age	− 0.850	0.286	− 0.134	0.003	− 5.264	0.412	− 0.615	** < 0.0001**
		Disease time	− 0.164	0.097	− 0.073	0.093	− 0.308	0.095	− 0.139	**0.001**
		Flexion CA	− 0.460	0.354	− 0.069	0.194	− 0.687	0.438	− 0.082	0.118
		Cervical ROM	− 0.077	0.276	− 0.014	0.781	− 0.027	0.331	− 0.004	0.936
	C6-7 HU value	Age	− 0.965	0.240	− 0.177	** < 0.0001**	− 4.906	0.331	− 0.658	** < 0.0001**
		Disease time	− 0.209	0.082	− 0.108	**0.011**	− 0.279	0.076	− 0.145	** < 0.0001**
		Flexion CA	− 0.818	0.297	− 0.142	**0.006**	− 0.892	0.351	− 0.122	**0.012**
		Cervical ROM	− 0.041	0.232	− 0.009	0.860	− 0.139	0.266	− 0.025	0.601
	C2-7 HU value	Age	− 0.888	0.263	− 0.151	**0.001**	− 5.144	0.377	− 0.637	** < 0.0001**
		Disease time	− 0.179	0.090	− 0.086	**0.047**	− 0.298	0.087	− 0.143	**0.001**
		Flexion CA	− 0.065	0.255	− 0.013	0.077	− 0.755	0.400	− 0.096	0.060
		Cervical ROM	0.208	0.247	0.039	0.799	− 0.064	0.302	− 0.011	0.833
< 50	C2-5 HU value	Age	− 0.425	0.878	− 0.039	0.629	− 5.732	1.472	− 0.392	** < 0.0001**
		Disease time	0.184	0.195	0.079	0.344	− 0.084	0.174	− 0.050	0.630
		Flexion CA	0.056	0.653	0.009	0.931	0.018	0.796	0.003	0.982
		Cervical ROM	0.207	0.469	0.045	0.659	− 0.304	0.584	− 0.070	0.604
	C6-7 HU value	Age	− 1.049	0.726	− 0.116	0.150	− 4.968	1.247	− 0.400	** < 0.0001**
		Disease time	0.037	0.161	0.019	0.820	0.032	0.148	0.032	0.826
		Flexion CA	− 0.274	0.540	− 0.052	0.613	− 0.140	0.675	− 0.027	0.836
		Cervical ROM	0.169	0.388	0.044	0.663	− 0.353	0.495	− 0.096	0.477
	C2-7 HU value	Age	− 0.633	0.809	− 0.022	0.435	− 5.478	1.365	− 0.404	** < 0.0001**
		Disease time	0.135	0.179	0.063	0.452	− 0.045	0.162	− 0.029	0.779
		Flexion CA	− 0.054	0.602	− 0.009	0.929	− 0.035	0.738	− 0.006	0.963
		Cervical ROM	0.195	0.432	0.046	0.653	− 0.320	0.542	− 0.079	0.556
50–60	C2-5 HU value	Age	− 0.748	1.951	− 0.030	0.702	− 10.640	2.376	− 0.395	** < 0.0001**
		Disease time	0.039	0.157	0.020	0.801	− 0.343	0.162	− 0.179	**0.036**
		Flexion CA	− 0.651	0.685	− 0.090	0.344	− 0.800	0.770	− 0.100	0.301
		Cervical ROM	− 0.346	0.555	− 0.059	0.533	− 0.275	0.578	− 0.046	0.635
	C6-7 HU value	Age	− 0.993	1.585	− 0.002	0.545	− 7.560	1.919	− 0.341	** < 0.0001**
		Disease time	− 0.024	0.132	− 0.014	0.853	− 0.390	0.131	− 0.247	**0.003**
		Flexion CA	− 0.868	0.575	− 0.142	0.133	− 1.305	0.622	− 0.198	**0.038**
		Cervical ROM	− 0.295	0.466	− 0.060	0.527	− 0.379	0.467	− 0.077	0.418
	C2-7 HU value	Age	− 0.829	1.797	− 0.036	0.645	− 9.613	2.175	− 0.386	** < 0.0001**
		Disease time	0.018	0.363	− 0.286	0.900	− 0.359	0.148	− 0.202	**0.017**
		Flexion CA	− 0.723	0.631	− 0.108	0.254	− 0.969	0.705	− 0.131	0.172
		Cervical ROM	− 0.329	0.511	− 0.061	0.520	− 0.310	0.529	− 0.056	0.559
> 60	C2-5 HU value	Age	− 0.807	0.908	− 0.060	0.375	− 0.139	1.073	− 0.013	0.897
		Disease time	− 0.678	0.172	− 0.273	** < 0.0001**	− 0.361	0.151	− 0.220	**0.019**
		Flexion CA	− 0.464	0.547	− 0.067	0.398	− 1.117	0.668	− 0.184	0.097
		Cervical ROM	0.111	0.445	0.020	0.803	0.761	0.528	0.157	0.153
	C6-7 HU value	Age	− 0.102	0.769	− 0.009	0.895	− 1.593	0.784	− 0.185	**0.045**
		Disease time	− 0.587	0.145	− 0.275	**< 0.0001**	− 0.365	0.111	− 0.285	**0.001**
		Flexion CA	− 0.984	0.463	− 0.167	**0.035**	− 1.072	0.488	− 0.226	**0.030**
		Cervical ROM	0.177	0.377	0.037	0.639	0.615	0.386	0.163	0.114
	C2-7 HU value	Age	− 0.572	0.837	− 0.046	0.495	− 0.624	0.951	− 0.063	0.513
		Disease time	− 0.648	0.158	− 0.281	** < 0.0001**	− 0.362	0.134	− 0.245	**0.008**
		Flexion CA	− 0.637	0.504	− 0.100	0.208	− 1.102	0.592	− 0.201	0.065
		Cervical ROM	0.133	0.410	0.0269	0.746	0.712	0.468	0.163	0.131

Bold indicates statistically significant difference,* P* < 0.05

Adjusted for age (not for age subgroup analyses), BMI, neck pain, comorbidities and disease type. Bold values indicate statistically significant. BMI, neck pain, comorbidities and disease type data are not shown

## Discussion

In this retrospective study, we clarify the age-related influences of disease time, cervical alignment and ROM on the cervical HU value. Importantly, cervical HU value was significantly correlated with the disease time, flexion CA and cervical ROM. In addition, disease time and flexion CA were negatively affecting the C6-7 HU value in more than 60-year-old males and in more than 50-year-old females.

Our results found that cervical vertebral HU value varies by level, C2-C5 HU values are maintained at a baseline, the decrease was obvious at C6 and the C7 vertebral had the minimum HU value. Our cervical HU values are similar to previous investigations in the cervical spine [18, 19]. Francis et al. [18] examined 201 cervical spines CTs and reported mean C2-C7 HU values remarkably similar to ours, suggesting the lowest HU scores in the lower cervical spine, which may partially explain the high rate of mechanical complications in multilevel anterior fusions, because the BMD of the cervical is closely related to perioperative complications and patients with osteoporosis were more likely to undergo revision surgery with implant failure [20]. Our study divided cervical vertebral HU value into upper cervical vertebral (average HU value of C2-5 vertebral), lower cervical vertebral (average HU value of C6-7 vertebral) and overall cervical vertebral (C2- C7 vertebral average HU value). It is better to study the influence of different factors on cervical BMD according to bone characteristics.

The effect of age and sex on bone quality has been confirmed by many studies [5, 21]. We found that cervical HU value significantly decreased with age, especially in females. Our results suggest that the less than 40 age group had no statistical difference compared to the 40–50 age group, between 60 and 70 age group and the more than 70 age group also had no statistical difference, and the 50–60 age group was significantly different compared to less than 50 and more than 60 age groups. Females lose more bone during perimenopause, in less than 50 age groups, and males had lower total HU values than females, whereas, in the 50–60 age group, HU values in males started to be higher than in females, especially in C6-7. In more than 60 age groups, males had higher total HU values than females. Similar results were reported in previous investigations [4, 22, 23]. Therefore, according to age, we performed subgroup analysis to analyze the impact of disease time, flexion CA and cervical ROM on cervical HU value.

Our study showed that cervical HU values decreased with increased disease time and there were weak but significant negative correlations. Multivariate analyses suggest that the C6-7 HU value was affected by disease time. Gou et al. [24] suggest that the duration of symptoms was an independent risk factor for poor postoperative efficacy in patients with degenerative cervical myelopathy. Another study showed that inter-vertebral disk degeneration is related to osteopenia of adjacent vertebrae [25]. We think it may be due to the influence of the cervical on the mechanical duration of the pathological state and the disease time of long-term cervical loading may affect bone mineral density.

Our correlation analysis demonstrated that flexion CA and cervical ROM were significantly associated with the cervical HU value, especially in the C6-7 HU value. A mechanical study showed that compression increases cranially to caudally throughout the cervical spine. Conversely, in a neutral posture, there is a constant anterior shear at each joint level. Compression increased twofold throughout the cervical with flexion [26]. In response to these different long-term loading patterns, the bone tissue adapts among vertebral levels. BMD may be influenced to a greater extent by the type and direction of shear, torsion and bending loading. Clinical studies indicate that the incidence of disk degeneration was most severely seen at C5/C6 and C6/C7 disks and significantly increased with age [27]. Another study showed that cervical flexion is associated with neck pain worse, and the patient may avoid flexion to reduce pain resulting in decreased flexion [28]. Taken together, our findings confirm the effect of flexion CA on the C6-7 HU value, especially in more than 60-year-old males and in more than 50-year-old females.

This study has several limitations. First, this study did not consider all clinical factors (BMI, ethnicity, smoking, etc.), and patient symptoms such as neck pain and neck stiffness were not available, so additional studies are necessary. Second, we did not conduct a DXA examination, which is no proven association between DXA and HU values in the present study. Third, the number of subjects aged less than 30 and more than 80 years was small, and the results in the age population may be less accurate in those age groups. Fourth, we collected disease time from our hospital's case data, and some patients' symptoms may not be persistent and disease severity not be quantified. Fifth, the upper and lower cervical was anatomically complex and there may be other influencing factors that affect the HU value of the cervical. Last but not least, in this study, the HU value and clinical data were obtained from a single hospital, and prospective multicenter investigations are further needed to account for other factors to extend our results.

In summary, we found that disease time and flexion CA were negatively affecting the C6-7 HU values in more than 60-year-old males and in more than 50-year-old females. Therefore, surgeons should pay more attention to bone quality in those patients with longer disease time and larger convex of flexion CA.

## Data Availability

The datasets and materials supporting the conclusions of this article are included within the article, and further inquiries can be directed to the corresponding author.

## Abbreviations

ROM: Range of motion
HU: Hounsfield unit
CT: Computed tomography
TMC: Titanium mesh cage
BMD: Bone mineral density
DXA: Dual-energy X-ray absorptiometry
ROI: Region of interest
PACS: The picture archiving and communication system
CA: C2-7 Cobb angle
